# Negative biotic interactions drive predictions of distributions for species from a grassland community

**DOI:** 10.1098/rsbl.2018.0426

**Published:** 2018-11-14

**Authors:** Phillip P. A. Staniczenko, K. Blake Suttle, Richard G. Pearson

**Affiliations:** 1 National Socio-Environmental Synthesis Center (SESYNC), Annapolis, MD, USA; 2 Department of Biology, University of Maryland College Park, College Park, MD, USA; 3 Department of Ecology and Evolutionary Biology, UC Santa Cruz, Santa Cruz, CA, USA; 4 Centre for Biodiversity and Environment Research, University College London, London, UK

**Keywords:** species geographical ranges, species distribution models, biotic interactions, model selection, normalized maximum likelihood, minimum description length principle

## Abstract

Understanding the factors that determine species' geographical distributions is important for addressing a wide range of biological questions, including where species will be able to maintain populations following environmental change. New methods for modelling species distributions include the effects of biotic interactions alongside more commonly used abiotic variables such as temperature and precipitation; however, it is not clear which types of interspecific relationship contribute to shaping species distributions and should therefore be prioritized in models. Even if some interactions are known to be influential at local spatial scales, there is no guarantee they will have similar impacts at macroecological scales. Here we apply a novel method based on information theory to determine which types of interspecific relationship drive species distributions. Our results show that negative biotic interactions such as competition have the greatest effect on model predictions for species from a California grassland community. This knowledge will help focus data collection and improve model predictions for identifying at-risk species. Furthermore, our methodological approach is applicable to any kind of species distribution model that can be specified with and without interspecific relationships.

## 1. Introduction

Species' distributions are commonly estimated using only abiotic environmental variables, but recent studies have shown that also modelling biotic interactions can improve range predictions [1]. Because ecological complexity makes describing all interspecific relationships in a community practically impossible, it will be useful to know which types of interaction (e.g. facilitation, competition) are priorities for data collection and modelling. A practical starting point for this effort is identifying which interspecific relationships have the greatest influence on geographical range predictions in previously documented communities.

Here we use information theory [2] to measure the effect of interspecific relationships on the distributions of species from a well-documented California grassland community [3,4]. The general idea of information theory is that models compress data, and better models compress data by larger amounts. In this application, we represent different types and combinations of interspecific relationships by Bayesian network (BN) models and measure how much each model compresses data on multiple-species distributions. We find that negative biotic interactions are the main driver of species distributions. This result suggests prioritizing field studies involving competition and theories about niche overlap to inform range predictions. Modelling facilitation and shared habitat suitability relationships is still valuable, but may result in less pronounced adjustments to species' distributions.

## 2. Material and methods

### (a) Generating community distribution matrices

**Figure 1.**
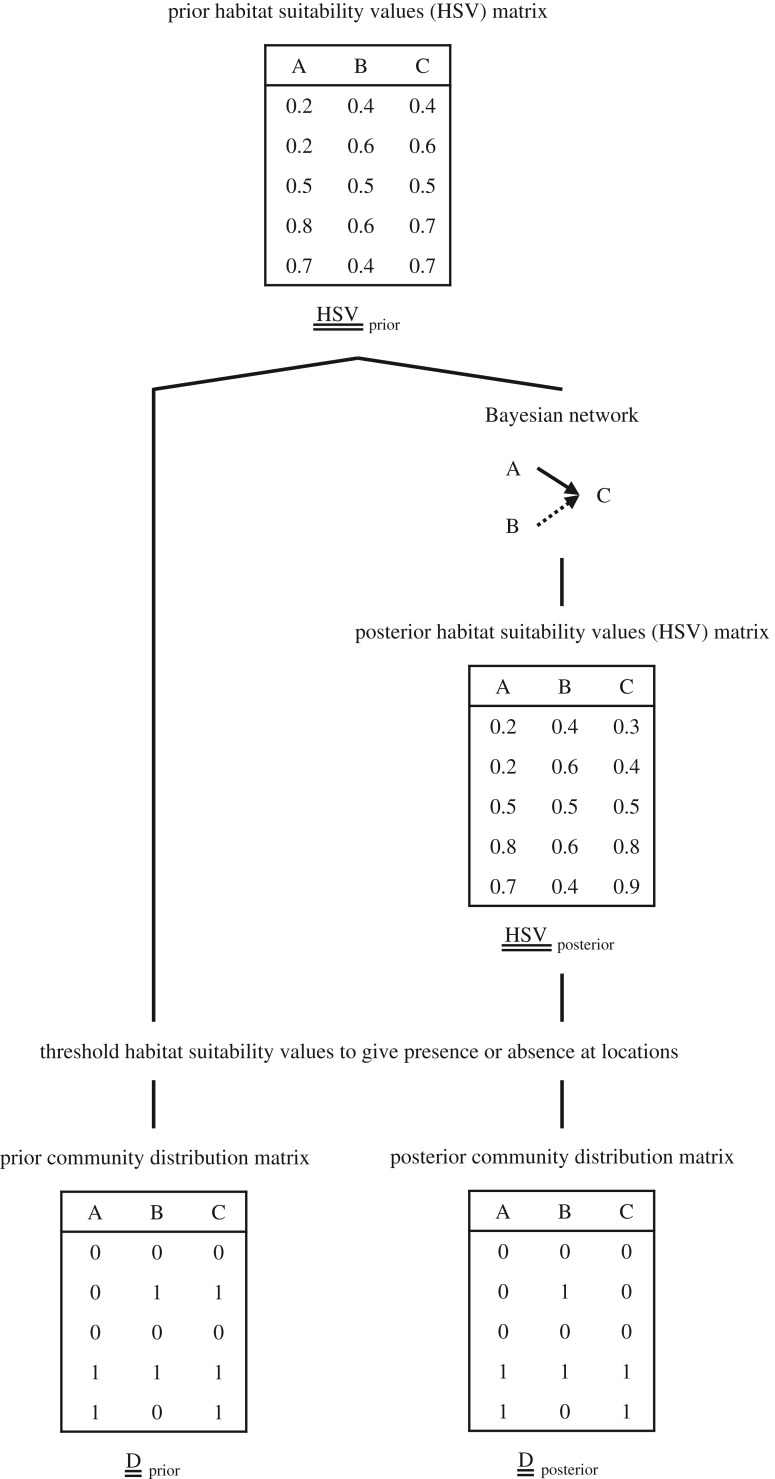
Workflow for generating community distribution matrices. The starting point is a matrix of prior habitat suitability values (HSVs) that reflect only abiotic conditions for each species (columns) at distinct locations (rows). We then use a Bayesian network to modify prior HSVs to give posterior HSVs that also include the effects of interspecific relationships on species distributions. For each species and both HSV matrices separately, we specify thresholds to convert prior and posterior HSVs to binary ranges.

We refer to a collection of geographical range predictions in the same region as a ‘community distribution’ and a community distribution matrix describes binary range predictions for multiple species (columns) at distinct locations (rows). To be clear, the matrix does not represent the range of a community as a whole, but rather the ranges of individual species together as a meta-community. We consider two versions for analysis: a *prior* community distribution matrix based on species distribution models (SDMs) *without* interspecific relationships, and a *posterior* community distribution matrix based on SDMs *with* interspecific relationships (figure 1).

For the prior community distribution matrix, we combined presence records for 54 species and seven bioclimate variables to determine favourable habitat conditions for each species following the Maxent method [5], and used Maxent's logistic output as a habitat suitability value at each location [6] (see electronic supplementary material). This environment-only approach provided good baseline ranges for species from the California grassland community [1].

For the posterior community distribution matrix, we used the same Maxent parameter estimates for species' responses to bioclimate variables as above but also modelled the effects of 52 interspecific relationships (classified from experiments [3] and long-term monitoring studies [4]; see electronic supplementary material for results for two alternative sets of interspecific relationships) on 14 focal species using a method that has been shown to improve range predictions for these species [1]. This earlier study used BNs to modify prior habitat suitability values to generate posterior habitat suitability values (unlike the present study which uses BNs to assess the strength of similarity or difference between range predictions for different species), but any SDM method that includes the effects of interspecific relationships can be used to generate a posterior community distribution matrix.

For each focal species, we specified a threshold for converting habitat suitability values to a binary range, with potentially different thresholds for prior and posterior values. We considered two rules for determining thresholds: (i) the habitat suitability value that maximizes classification sensitivity and therefore results in all presence records for a species being included in its estimated range (maxSens threshold); and (ii) the habitat suitability value that maximizes both classification sensitivity and specificity (maxSSS threshold; [7]).

### (b) Comparing the effect of interspecific relationships on community distributions

We considered a suite of eight compression models to explain range predictions (table 1). The suite contained a model with all 52 interspecific relationships (ALL) used to generate the posterior community distribution matrix, and subsets representing all combinations of three types of interspecific relationship: positive biotic interactions (BI+), negative biotic interactions (BI−), and shared habitat suitability (SHS; although not a biotic interaction, environmental co-responses can be measured and included in models to maximize the usefulness of empirical data [1]).

**Table 1.** Model performance with all interspecific relationships (ALL) and subsets of positive and negative biotic interactions (BI) and shared habitat suitability (SHS); absolute changes, Δ_M_, are rescaled such that ±1 is the number of bits required to transmit an uncompressed community distribution matrix.

	model	#positive	#negative	Δ_M_	rank	Δ%_M_	rank
maxSens threshold	ALL	40	12	−0.008	4	−2.8%	3
	SHS BI	38	9	−0.011	6	−3.8%	5
	SHS BI+	38	0	−0.016	8	−5.9%	7
	SHS BI−	32	9	−0.011	5	−3.8%	6
	SHS	32	0	−0.016	7	−6.2%	8
	BI	6	9	0.003	2	3.7%	2
	BI+	6	0	−0.001	3	−3.2%	4
	BI−	0	9	0.005	1	9.7%	1
maxSSS threshold	ALL	40	12	0.041	1	16.1%	2
	SHS BI	38	9	0.037	2	14.7%	4
	SHS BI+	38	0	0.029	4	12.5%	6
	SHS BI−	32	9	0.035	3	15.8%	3
	SHS	32	0	0.028	5	13.7%	5
	BI	6	9	0.008	6	9.6%	7
	BI+	6	0	−0.001	8	−1.2%	8
	BI−	0	9	0.007	7	20.9%	1

Interspecific relationships are represented as conditional dependencies in a BN model. These conditional dependencies assume that linked species have non-independent ranges, so the BN model can be used to assess how well a given set of interspecific relationships explains the particular pattern of ranges in a community distribution matrix. For example, the BN in figure 1 is designed to test if the predicted ranges of species A and B contain meaningful information about the predicted range of species C. Because BNs are acyclic, the direction of a conditional dependency must be specified even when the effects of an interaction are symmetric, which places limitations on how well interspecific interactions can be described using this approach (see electronic supplementary material).

We used *total length* to measure the amount of data compression resulting from each model because, as with related statistical estimators used in model selection such as the Akaike information criterion, it accounts for both model fit and complexity [8]. Total length has a straightforward interpretation as the number of *bits* required to describe a data-model combination, with the most parsimonious model for a given dataset resulting in the shortest total length [9].

**Figure 2.**
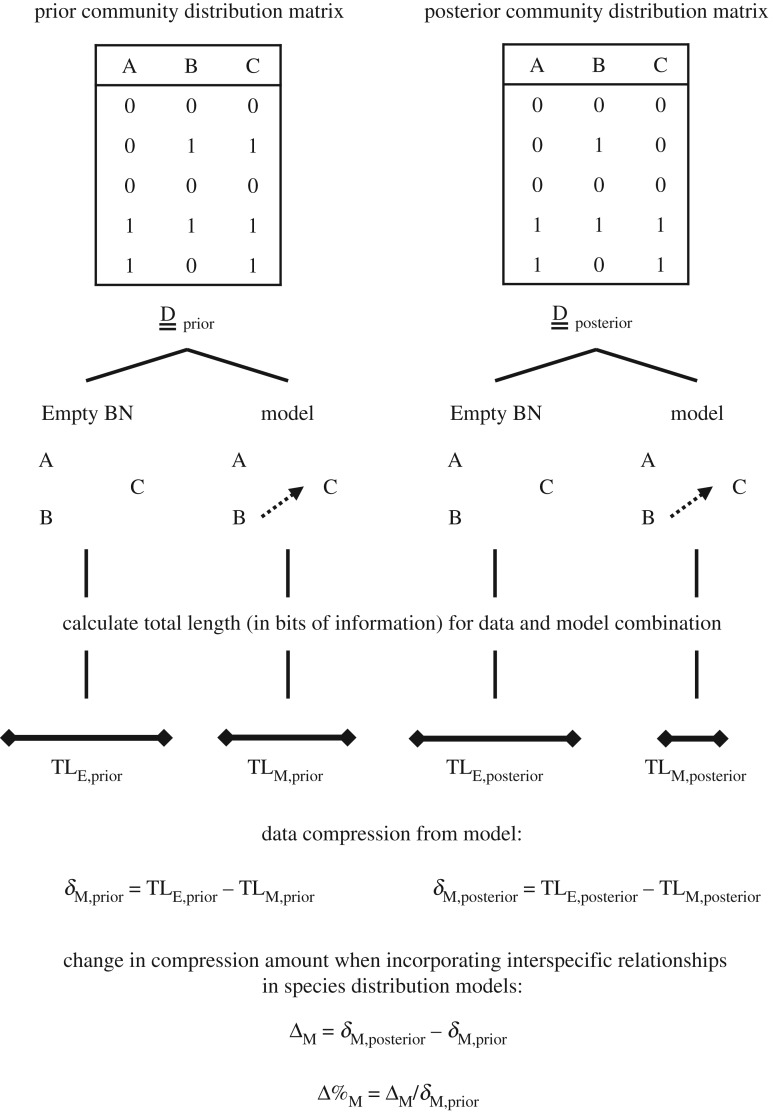
Workflow for measuring the effect of interspecific relationships on community distribution matrices. For each prior and posterior matrix, we calculate total length for a compression model with no interspecific relationships (‘Empty BN’), and total length for a compression model, M, representing a subset of the interspecific relationships used to generate the posterior community distribution matrix. A particular subset of interspecific relationships can be said to significantly influence range predictions if model M compresses the posterior community distribution matrix more than the Empty BN, and the increase in compression is greater than the comparable increase with the prior community distribution matrix.

We assessed the effect of including a particular subset of interspecific relationships in SDMs by comparing differences in total length between a pair of prior and posterior community distribution matrices for the same BN model (figure 2). We began by calculating total lengths for the two matrices and a BN model representing no interspecific relationships: TL_E,prior_ and TL_E,posterior_. Such ‘Empty BN’ models have no conditional dependencies among species and provide a baseline measurement of the amount of information complexity inherent in a community distribution matrix. For example, very little information is required to describe a community distribution matrix in which all species are present (or absent) at all locations.

Then for each BN model, M, under consideration, we calculated one total length for the prior community distribution matrix, TL_M,prior_, and another for the posterior community distribution matrix, TL_M,posterior_. We obtained comparable values for the amount of compression resulting from the model by calculating the change in total length from the Empty BN for the two community distribution matrices separately:2.1
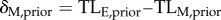
and2.2
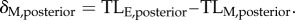


The effect of including a particular subset of interspecific relationships in SDMs can be expressed as the absolute change in data compression associated with model M:2.3
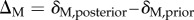
and as a percentage change:2.4
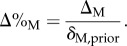


Equations (2.3) and (2.4) ensure we isolate the additional compression effect of including particular interspecific relationships in SDMs, over and above range similarities (or differences) due to correlated (or anti-correlated) environmental responses among species. In general, absolute changes will be smaller for BN models that contain fewer conditional dependencies because not all species ranges will be compressed. This means percentage changes are useful for investigating which types of interspecific relationships provide the most compression relative to their preponderance.

## 3. Results

When using the maxSens threshold to convert habitat suitability values to binary ranges, only two of the eight models resulted in positive changes in data compression (i.e. Δ_M_ > 0): the model representing nine negative biotic interactions led to a percentage change in total length of Δ%_BI−_ = 9.7%; and the model representing all 15 positive and negative biotic interactions led to Δ%_BI_ = 3.7% (table 1).

With the maxSSS threshold, seven of the eight models resulted in positive changes in data compression. All absolute changes in total length, Δ_M_, for the same model were larger than with the maxSens threshold. The ALL model led to the largest absolute change, and the rank order of models followed the number of interspecific relationships represented in compression models; the single negative change resulted from the model representing six positive biotic interactions. The model representing negative biotic interactions led to the largest percentage change in total length: Δ%_BI–_ = 20.9%; followed by the ALL model with Δ%_ALL_ = 16.1%. The third largest percentage change was for the compression model representing both negative biotic interactions and shared habitat suitability relationships: Δ%_SHS BI−_ = 15.8%.

These results show that negative biotic interactions drive predicted ranges. There are three notable examples of competitive effects in the grassland system [1,3,4]: direct competition between annual grasses and winter forbs, indirect competition due to annual grass litter inhibiting the germination and growth of spring and summer forbs, and competition among perennial grasses and summer forbs for limited water resources.

## 4. Discussion

Our findings suggest that processes like competitive exclusion have the greatest influence on predicted distributions once abiotic factors have been taken into account. Interestingly, compression models representing shared habitat suitability relationships performed relatively poorly. So, although many studies report a predominance of positive associations among species [10–12], modelling such relationships in SDMs might not have as discernible an effect on predicted species distributions as negative associations. As such, we recommend focusing data collection in regions where the predicted ranges of competing species overlap, to help further parameterize the effect of competition on species that would otherwise be expected to co-occur frequently.

It remains an open question under what circumstances and at which spatial scales species affect one another's ranges. Recent work suggests that negative associations among species are more likely to be observed at small spatial scales and fine resolutions, whereas positive associations are expected to dominate at large spatial scales and coarse resolutions [13]. Our method offers a novel and tractable way to test this hypothesis, first, by comparing results for similar ecological communities at a variety of spatial scales, and second, by looking for consistent patterns across different biological systems. A promising extension when joint observation data (i.e. when two or more species are recorded as present at a single location) are available involves using joint SDMs [14] that leverage environmental co-responses among species to set a higher bar for establishing the predictive value of biotic interactions.

SDMs are commonly used in extinction risk assessments, with large range losses under environmental change indicating at-risk species. As SDMs continue to include more than just abiotic variables, it will be important to consider not only which interspecific relationships are worth modelling now, but also how those relationships will change in the future. We hope this approach inspired by information theory will help clarify how biotic interactions shape community distributions, and guide efficient strategies for improving range predictions and extinction risk assessments.

## Supplementary Material

Additional description of materials and methods

## Data accessibility

All data are publically available and described in electronic supplementary material. Code to perform species distribution modelling is freely available as an accompaniment to [1]. Code to perform total length analysis is available at: http://dx.doi.org/10.5061/dryad.7h4001r [15].
