# Supplementary material for: Negative biotic interactions drive predictions of distributions for species from a grassland community
Source: Biol Lett. 2018 Nov 14;14(11):20180426. doi: 10.1098/rsbl.2018.0426 (PMC6283927; doi:10.1098/rsbl.2018.0426)
Supplement: Additional description of materials and methods [file rsbl20180426supp1.pdf]

SUPPLEMENTARY INFORMATION

Negative biotic interactions drive predictions of distributions for species from a grassland community

Phillip P.A. Staniczenko[1,2,†], K. Blake Suttle[3] & Richard G. Pearson[4]

[1]National Socio-Environmental Synthesis Center (SESYNC), MD, USA

[2]Department of Biology, University of Maryland College Park, MD, USA

[3]Department of Ecology and Evolutionary Biology, UC Santa Cruz, CA, USA

[4]Centre for Biodiversity and Environment Research, University College London, UK

[†]Present address: Department of Biology, Brooklyn College, City University of New York, NY, USA

Author for correspondence: Phillip P.A. Staniczenko, phillip.staniczenko@brooklyn.cuny.edu

This document contains additional description of materials and methods in two sections: (i) Species distribution models for the California grassland community, and (ii) Measuring data compression using Bayesian networks and total length.

Species distribution models for the California grassland community

We implemented species distribution models (SDMs) in R using the BIOMOD platform (Thuiller *et al.* 2009). The species pool contained 53 plant and 1 grasshopper species (Table S1) found at the Angelo Coast Range Reserve (39° 44′ 17.7″ N, 123° 37′ 48.4″ W), a protected and relatively undisturbed area of 7,660 acres in northern California. We downloaded presence records for these species from the Global Biodiversity Information Facility (http://GBIF.org), and, for each species, we classified as "present" any 800m × 800m grid cell in the western USA (32° N to 49.1° N and 125° W to 115° W) that contained one or more presence records.

For both prior and posterior community distribution matrices, we used the Maxent method (Elith *et al.* 2011) to estimate species' responses to bioclimate variables. Following Fithian & Hastie (2013), consider predicting the distribution of an individual species across a geographical domain $\mathcal{D}$ at locations $z \in \mathcal{D}$. Associated with each location is a vector of measured bioclimate variables or features $x(z)$. A presence-only data set consists of n_1 locations of species sightings $z_k \in \mathcal{D}$ for $k = 1, 2, \ldots, n_1$, and n_0 "background" locations z_k for $k = n_1 + 1, 2, \ldots, n_1 + n_0$; and let $x_k = x(z_k)$ be the features for location k. The suitability of a location for the species is given by $N(z_k) \sim \text{Poisson}(\lambda(z_k))$; where the intensity is log-linear in features $x(z)$:

$$\lambda(z) = e^{\alpha + \beta x(z)} \tag{S1}$$

Notice that α only multiplies $\lambda(z)$ by a constant, so β completely determines the distribution of habitat suitability across the geographical domain: $p_\lambda(z) = \frac{e^{\beta x(z)}}{\int_\mathcal{D} e^{\beta x(z)} dz}$. Computing the maximum entropy solution is equivalent to computing the maximum likelihood for Eqn (S1) given sighting data. There are two steps: i) Obtain the maximum likelihood estimate density $\hat{p}_\lambda(z)$, which requires finding $\hat{\beta}$ for which the density is "nearly geographically uniform" (i.e.,

entropy is maximised) subject to constraints that make it resemble the sighting data; and ii) Multiply $\hat{p}_\lambda(z)$ by n_1, which requires finding $\hat{\alpha}$ such that $\hat{\lambda}(z) = n_1\hat{p}_\lambda(z)$. In practice, we used Maxent's logistic output in BIOMOD to obtain prior habitat suitability values (ranging from 0 to 1) at each location.

We considered seven bioclimate variables: maximum temperature of the warmest month; minimum temperature of the coldest month; annual precipitation; precipitation of the driest quarter; mean temperature of the wettest quarter; temperature seasonality (standard deviation $\times$ 100); and precipitation seasonality (coefficient of variation). The climate variables were derived from an ensemble of five atmosphere-ocean general circulation models for the year 2010, and are freely available online (Pearson *et al.* 2014; http://dx.doi.org/10.7917/D7WD3XH5). For reference, climate variables at Angelo Coast Range Reserve are provided in Table S2.

For the posterior community distribution matrix, we modelled the effects of biotic interactions, shared habitat suitability, and other interspecific relationships on range predictions for 14 focal species using a method that also uses Bayesian networks (BNs; Staniczenko *et al.* 2017). The California grassland BN contains a total of 52 conditional dependencies, representing 6 positive and 9 negative biotic interactions, 32 positive shared habitat suitability relationships, and 2 positive and 3 negative uncategorised interspecific relationships; these interspecific relationships were classified according to a series of transplant and competitor-removal experiments (Thomsen *et al.* 2006; Suttle & Thomsen 2007), consumer addition and removal experiments (Suttle *et al.* 2007), and long-term monitoring studies (Sullivan *et al.* 2016). In practice, conditional dependencies modify prior habitat suitability values to produce posterior habitat suitability values that also include the effects of interspecific relationships; as with our prior habitat suitability values, posterior habitat suitability values

also range from 0 to 1. Because prior habitat suitability values for non-focal species were, by design, unchanged by the California grassland BN, we only considered the 14 focal species in subsequent analysis.

Given a set of prior habitat suitability values for the 14 focal species and a corresponding set of posterior habitat suitability values, we converted habitat suitability values for each species in each matrix to a binary range using one of two threshold rules. For example, with the maxSens threshold, we considered each species in turn and identified the habitat suitability value that would result in all recorded presences (species sightings) being included in its estimated range; that is, all habitat suitability values above the threshold value were assigned "1" and all habitat suitability values below the threshold value were assigned "0". A similar process was used for the maxSSS threshold, but a different, typically larger threshold habitat suitability value was identified for each species and each matrix. The resulting prior and posterior community distribution matrices describe binary range predictions for multiple species (columns) at distinct locations (rows), and are suitable for data compression analysis to determine which interspecific relationships drive predictions of species' distributions.

Measuring data compression using Bayesian networks and total length

In data compression analysis, we represent interspecific relationships by conditional dependencies in a BN model. Whereas in the above section we used BNs to modify prior habitat suitability values to generate posterior habitat suitability values, we now use BNs to assess the strength of similarity or difference between range predictions in a community distribution matrix. For example, consider the BN model in Fig. 1 of the main text, which represents three species and one positive and one negative interspecific relationship by three nodes and

one positive and one negative conditional dependency: A $\rightarrow$ C and B $\not\rightarrow$ C. Rather than prescribing a rule that increases the habitat suitability value of C if A is likely to be present at a particular location and decreasing the habitat suitability value of C if B is likely to be present at a particular location, in this application we use the BN as a compression model to test if the predicted ranges of species A and B contain meaningful information about the predicted range of species C.

In this example, we do not need to consider state tables (also known as conditional probability tables) for species A and B for data compression analysis because their binary ranges are not affected by interspecific relationships—in SDMs, at least—and are therefore the same in both prior and posterior community distribution matrices. The state table for species C is

A	B	C
1	1	$p_{\mathrm{C},3}$
1	0	$p_{\mathrm{C},2}$
0	1	$p_{\mathrm{C},1}$
0	0	$p_{\mathrm{C},0}$

where there is a distinct probability for each unique state of A and B. We would expect $p_{\mathrm{C},2}$ to have the highest value given the assumption of a positive conditional dependency between A and C, $p_{\mathrm{C},1}$ to have the lowest value given the assumption of a negative conditional dependency between B and C, and intermediate values for $p_{\mathrm{C},0}$ and $p_{\mathrm{C},3}$ (whether $p_{\mathrm{C},0} \geq p_{\mathrm{C},3}$ or $p_{\mathrm{C},0} \leq p_{\mathrm{C},3}$ would be determined by how A and B combine to affect C).

Maximum likelihood probabilities are calculated from data, in this case community distribution matrices, and for data compression analysis their relative values are less important than the requirement that each state has a distinct probability (the is equivalent to the "FULL" model described in Staniczenko *et al.* 2017). This requirement ensures that total

lengths, which are calculated from maximum likelihood probabilities, reflect not only the effects of each interspecific relationship on range predictions, but also the effects of each *combination* of interspecific relationships on range predictions.

Total length can be calculated as the $\log_2$-transformed version of normalized maximum likelihood (NML; Rissanen 2001; Myung *et al.* 2006; Staniczenko *et al.* 2014). In this study, NML quantifies how well a BN model representing interspecific relationships explains a particular community distribution matrix compared to how well the model explains *all possible* community distribution matrices with the same number of species and locations. A good model explains only the focal community distribution matrix well and all other matrices poorly, resulting in high NML and short total length. By contrast, an overly simplistic model is likely to explain the focal matrix no better than it does any other matrix, resulting in low NML and long total length. And although an overly complex model is likely to explain the focal matrix well, it will also explain all other matrices well due to its greater inherent flexibility, still resulting in low NML and short total length.

For BN model M and community distribution matrix $\mathcal{B}$, NML can be written as

$$\mathrm{NML}(M|\mathcal{B}) = \frac{\hat{L}(M, \vec{\hat{p}}_{\mathcal{B}}|\mathcal{B})}{\sum_{\mathcal{B}'} \hat{L}(M, \vec{\hat{p}}_{\mathcal{B}'}|\mathcal{B}')} \tag{S2}$$

where $\hat{L}$ is a likelihood function and $\vec{\hat{p}}_{\mathcal{B}}$ is a vector of maximum likelihood probabilities from state tables associated with M. In Eqn (S2), conventional likelihood is normalised over all matrices $\mathcal{B}'$ with the same number of rows and columns as the original community distribution matrix $\mathcal{B}$.

For binary matrices, each element can be considered the outcome of a Bernoulli trial and so the maximum likelihood estimate of state table probability p_l is

$$\hat{p}_l = \frac{U_l}{U_l + Z_l} \tag{S3}$$

where U_l is the number of ones (locations within a predicted range) and Z_l the number of zeros (locations outside a predicted range) in $\mathcal{B}$ that are associated with p_l. For example, consider the state table for species C, and, in particular, the case in which the predicted ranges of species A and B overlap, which has an associated probability $p_{\mathrm{C},3}$. Imagine that a corresponding community distribution matrix has 20 rows (locations) and the predicted ranges of A and B overlap at 6 locations. If the predicted range of C includes 4 of these 6 locations then $\hat{p}_{\mathrm{C},3} = 4/(4+2) = 2/3$. Similar calculations involving sections of the other 14 rows (e.g., locations in which the predicted ranges of A and B do not overlap for $\hat{p}_{\mathrm{C},0}$) would give maximum likelihood estimates for the three remaining state table probabilities.

The maximum likelihood function for binary matrices is

$$\hat{L}(M, \vec{\hat{p}}_{\mathcal{B}} | \mathcal{B}) = \prod_l \hat{p}_l^{U_l} (1 - \hat{p}_l)^{Z_l} \tag{S4}$$

where the product is taken over the complete set of state table probabilities in $\vec{\hat{p}}_{\mathcal{B}}$.

Total length, as the $\log_2$-transformed version of NML, is

$$\begin{aligned}\mathfrak{L}_M(\mathcal{B}) &= -\log_2 \mathrm{NML}(M|\mathcal{B}) \\ &= -\log_2 \hat{L}(M, \vec{\hat{p}}_{\mathcal{B}}|\mathcal{B}) + \log_2 \sum_{\mathcal{B}'} \hat{L}(M, \vec{\hat{p}}_{\mathcal{B}'}|\mathcal{B}') \\ &= -\log_2 \hat{L}(M, \vec{\hat{p}}_{\mathcal{B}}|\mathcal{B}) + \log_2 \mathcal{C}(M, \mathcal{B})\end{aligned} \tag{S5}$$

where $\mathcal{C}(M, \mathcal{B})$ is a penalisation constant known as the *parametric complexity* or *regret* (Rissanen 1986, 1987, 1989, 1996).

Parametric complexity has a surprisingly simple form when model parameters are conditionally independent (Staniczenko *et al.* 2014)[1]. However, calculating parametric complexity is less straightforward with BN models due to conditional dependencies between random variables (Grünwald 2007). One solution is factorised NML (fNML), which has been shown to provide a good approximation to NML (Silander *et al.* 2008, 2009). For BN models, fNML involves calculating NML for each matrix column separately and multiplying the results. In practice, Eqn (S5) is applied to each matrix column and corresponding state table in turn, and component total lengths are summed to give a final total length.

[1]Parametric complexity can be decomposed into separate contributions for each state table probability in the model. Writing $X_l = U_l + Z_l$, the denominator in Eqn (S2) becomes

$$\sum_{\mathcal{B}'} \hat{L}(M, \vec{\hat{p}}_{\mathcal{B}'}|\mathcal{B}^{'}) = \sum_{l} \hat{L}(M, \hat{p}_l|\mathcal{B}^{'}) = \prod_{l} \sum_{k=0}^{X_l} \binom{X_l}{k} \left(\frac{k}{X_l}\right)^k \left(\frac{X_l - k}{X_l}\right)^{(X_l-k)} = \prod_{l} \mathcal{C}(X_l) \tag{S6}$$

with the contribution for each state table probability p_l written as

$$\mathcal{C}(X_l) = 1 + \frac{e^{X_l}\Gamma(X_l, X_l)}{X_l^{X_l-1}} \approx 1 + \left(\sqrt{X_l \frac{\pi}{2}} - \frac{1}{3} + \frac{\sqrt{2\pi}}{24\sqrt{X_l}} - \frac{4}{135 X_l} + \frac{\sqrt{2\pi}}{576\sqrt{X_l^3}} + \frac{8}{2835 X_l^2}\right) \tag{S7}$$

which can be computed quickly (Staniczenko *et al.* 2014).

Because absolute changes in total length can be unwieldy when a community distribution matrix contains a large number of locations, it is clearer to present values scaled by the number of bits required to transmit an uncompressed version of the matrix. With binary matrices, this reference total length is simply the number of matrix rows (locations) multiplied by the number of matrix columns (species).

Implications of the requirement that Bayesian networks are acyclic for modelling interspecific relationships in species distribution models

The requirement that a Bayesian network is acyclic restricts how the effect of an interspecific relationship can be modelled in our approach, particularly when the effects of an interaction could be symmetric (e.g., mutualistic or competitive interactions). In the case of competition, the main consideration is that one species must be identified as the stronger of the two competitors, with the stronger competitor influencing the occurrence probability (or habitat suitability value) of the weaker competitor. Once the direction of influence has been defined, a high occurrence probability (or habitat suitability value) for the stronger competitor is modelled as reducing the occurrence probability (or habitat suitability value) for the weaker competitor compared to its environment-only probability. Clearly, this imposed asymmetry is more realistic when interaction strengths are unequal between species and less realistic when strengths are more equal. Finally, it is worth noting that modelling an interaction in this way has no effect on the stronger competitor—due to the uni-directionality of a conditional dependency, only the estimated range of the weaker competitor is considered in data compression analysis.

Implications of the requirement that Bayesian networks are acyclic for comparing the effect of interspecific relationships on community distributions

We tested the sensitivity of our results to the directionality of interspecific relationships in compression models by repeating analyses with a new set of Bayesian networks in which the sign of interspecific relationships is maintained from corresponding original versions but the direction of influence is reversed. We considered the same prior and posterior community distribution matrices with a new set of eight compression models: ALL reverse, SHS BI reverse, SHS BI+ reverse, SHS BI– reverse, SHS reverse, BI reverse, BI+ reverse, BI– reverse.

It is first important to note that it is not informative to compare raw values of Δ_{M} and $\Delta\%_{\mathrm{M}}$ between the set of original compression models and the set of corresponding "reverse" models. This is because different numbers of species are potentially affected and so the scaling constant is different between the two sets of models, i.e., 13 focal species are assessed for compression with ALL (1 of the 14 focal species has no conditional dependencies), in comparison to 7 focal species and 23 non-focal species with ALL reverse. However, it is informative to compare differences in model *rankings* between the set of original compression models and the set of "reverse" compression models.

Rankings are similar between the two sets of compression models (Table S3). With the maxSSS threshold, the ranking for Δ_{M} among "reverse" Bayesian network models is exactly the same as with original models; rankings are similar for $\Delta\%_{\mathrm{M}}$, with BI– and BI– reverse top-ranked in their respective model sets. With the maxSens threshold, rankings are similar for Δ_{M}, and BI– and BI– reverse are again top-ranked in their respective model sets for

$\Delta\%_{\mathrm{M}}$. These findings show that our approach, results, and conclusions are insensitive to directionality in Bayesian network compression models, especially when using the maxSSS threshold and when considering model rankings for $\Delta\%_{\mathrm{M}}$. (Of course, directionality is important when modelling interspecific relationships in species distribution models, such that range predictions are modified for the intended species.)

Results for alternative Bayesian networks for modifying habitat suitability values in species distribution models

We repeated our workflow with two alternative Bayesian networks for modifying habitat suitability values in SDMs (see Fig. 1 in main text): (i) only shared habitat suitability relationships (32 positive conditional dependencies) and (ii) only biotic interactions (6 positive and 9 negative conditional dependencies). With only shared habitat suitability relationships in SDMs, we would expect, of our eight compression models (ALL, SHS BI, SHS BI+, SHS BI–, SHS, BI, BI+, BI–; see Fig. 2 in main text), BI, BI+, and BI– to perform worse than compression models that include SHS because those interactions are not included in the SDMs. By contrast, with only biotic interactions in SDMs, we would expect BI, BI+, and BI– to perform better than compression models that include SHS.

We have already established that modelling 52 interspecific relationships between species from the California grassland community using a Bayesian network improves range predictions for 13 focal species, with model performance measured as increases in AUC compared to when no interspecific relationships are modelled in SDMs (Staniczenko *et al.* 2017; Fig. S1). Model performance is still good for the two alternative Bayesian networks with only shared

habitat suitability relationships (Fig. S2) and only biotic interactions (Fig. S3), although understandably fewer predicted ranges are modified.

With only shared habitat suitability relationships in SDMs, results for the maxSens threshold show that the SHS compression model moves from being lowly ranked (when all 52 interspecific relationships are modelled) to top-ranked while BI, BI+, and BI– move from highly ranked to bottom-ranked, as expected (for both Δ_{M} and $\Delta\%_{\mathrm{M}}$; Table S4). Similarly, results for the maxSSS threshold show that all compression models that include SHS rank higher than BI, BI+, and BI–.

With only biotic interactions in SDMs, results for the maxSens threshold show that BI, BI+, and BI– remain top-ranked (compared to when all 52 interspecific relationships are modelled) and the order remains BI– then BI then BI+ (for both Δ_{M} and $\Delta\%_{\mathrm{M}}$). Results for maxSSS and Δ_{M} show that BI, BI+, and BI– move from bottom-ranked to top-ranked. Interestingly, although results for maxSSS and $\Delta\%_{\mathrm{M}}$ show that BI and BI+ move from lowly ranked to highly ranked, as expected, BI– moves from top-ranked to bottom-ranked.

The finding that BI– drops in ranking when modelling only biotic interactions in SDMs suggests that modelling negative biotic interactions is most important when the two species involved in the interaction are likely to occur together in the absence of other species that may influence the presence of the focal species. One can understand this interpretation as follows. With our current implementation of modelling interspecific relationships in SDMs, the effect of negative biotic interactions is very strong in the absence of positive shared habitat suitability relationships. That is, modelled negative biotic interactions reduce habitat

suitability values (HSVs) of affected species more often and by greater amounts than they otherwise would if all interspecific relationships were to be modelled. Because HSVs are systematically lower when mainly negative biotic interactions are modelled in SDMs, maxSSS "overcorrects" when transforming HSVs to a binary presence-absence range, and in doing so *dilutes* the effect of modelling negative biotic interactions in SDMs. This dilution effect explains why BI– does not lead to as much compression when modelling only biotic interactions in SDMs compared to modelling all interspecific relationships. (Note that maxSens does not appear to result in this dilution effect.)

To illustrate this dilution effect, consider a focal species A that is negatively influenced by a biotic interaction with species B and positively influenced by a shared habitat suitability relationship (or some other positive interspecific relationship) with species C. First consider the case in which both negative and positive interspecific relationships are modelled in the SDM for species A. When all three species are likely to occur together based on an environment-only SDM—i.e., all three species have high prior HSVs at a particular location—then the effects of the positive and negative interspecific relationship are modelled as cancelling one another out. When only species A and B are likely to occur together, the negative biotic interaction has a large effect on the posterior HSV of species A, which is picked up by the BI– compression model. By contrast, consider the case in which the positive interspecific relationship between species A and C is not modelled in the SDM for species A. The negative biotic interaction between species A and B "incorrectly" reduces the posterior HSV of species A when all three species are likely to occur together. Subsequently, maxSSS

"overcorrects" and identifies a threshold HSV for species A that is very different from both the threshold value for prior HSVs and posterior HSVs when all interspecific relationships are modelled in the SDM for species A. This overcorrection leads to a dilution of the effect of negative biotic interactions on the predicted range of species A when only biotic interactions are modelled in SDMs, and therefore gives rise to the result that BI– performs less well as a compression model.

References

Ehrlén, J. & Morris, W.F. (2015). Predicting changes in the distribution and abundance of species under environmental change. *Ecol. Lett.*, 18, 303–314.

Elith, J., Phillips, S.J., Hastie, T., Dudík, M., Chee, Y.E. & Yates, C.J. (2011). A statistical explanation of MaxEnt for ecologists. *Divers. Distrib.*, 17, 43–57.

Fithian, W. & Hastie, T. (2013). Finite-sample equivalence in statistical models for presence-only data. *Ann. Appl. Stat.*, 7, 1917–1939.

Grünwald, P.D. (2007). *The Minimum Description Length Principle.* MIT Press.

Myung, J.I., Navarro, D.J. & Pitt, M.A. (2006). Model selection by normalized maximum likelihood. *J. Math. Psychol.*, 50, 167–179.

Pearson, R.G. *et al.* (2014). Life history and spatial traits predict extinction risk due to climate change. *Nat. Clim. Change*, 4, 217–221.

Rissanen, J. (1986). Stochastic complexity and modeling. *Ann. Stat.*, 14, 1080–1100.

Rissanen, J. (1987). Stochastic complexity. *J. R. Stat. Soc. B*, 49, 223–239.

Rissanen, J. (1989). *Stochastic Complexity in Statistical Inquiry.* World Scientific Publishing.

Rissanen, J. (1996). Fisher information and stochastic complexity. *IEEE Trans. Inf. Theory*, 42, 40–47.

Rissanen, J. (2001). Strong optimality of the normalized ML models as universal codes and information in data. *IEEE Trans. Inf. Theory*, 47, 1712–1717.

Silander, T., Roos, T., Kontkanen, P. & Myllymäki, P (2008). Factorized normalized maximum likelihood criterion for learning Bayesian network structures. In: *Proceedings of the 4th European workshop on probabilistic graphical models (PGM-08).*

Silander, T., Roos, T. & Myllymäki, P (2009). Locally minimax optimal predictive modeling with Bayesian networks. In: *Proceedings of the 12th International Conference on Artificial Intelligence and Statistics (AISTATS-09).*

Staniczenko, P.P.A, Sivasubramaniam, P., Suttle, K.B. & Pearson, R.G. (2017). Linking macroecology and community ecology: Refining predictions of species distributions using biotic interaction networks. *Ecol. Lett.*, 20, 693–707.

Staniczenko, P.P.A., Smith, M.J. & Allesina, S. (2014). Selecting food web models using normalized maximum likelihood. *Methods Ecol. Evol.*, 5, 551–562.

Sullivan, M.J.P., Thomsen, M. & Suttle, K.B. (2016). Grassland responses to increased rainfall depend on the timescale of forcing. *Glob. Change Biol.*, 22, 1655–1665.

Suttle, K.B. & Thomsen, M. (2007). Climate change and grassland restoration in California: lessons from six years of rainfall manipulation in a north coast grassland. *Madrono*, 54, 225–233.

Suttle, K.B., Thomsen, M.A. & Power, M.E. (2007). Species interactions reverse grassland responses to changing climate. *Science*, 315, 640–642.

Thomsen, M., D'Antonio, C., Suttle, K.B. & Sousa, W.P. (2006). Ecological resistance, seed density, and their interaction determine patterns of invasion in a California coastal grassland. *Ecol. Lett.*, 9, 160–170.

Thuiller, W., Lafourcade, B., Engler, R. & Araújo, M.B. (2009). BIOMOD – a platform for ensemble forecasting of species distributions. *Ecography*, 32, 369–373.

Table S1: List of species and number of occupied grid cells in the western USA extent (which covers a total of 1,308,071 grid cells). Highlighted rows are the 14 focal species with the most occupied cells.

Species ID	Name	Family	Functional group	#Occupied cells
1	*Achillea millefolium*	Asteraceae	Perennial forb	94
2	*Aira caryophyllea*	Poaceae	Annual grass	45
3	*Aphanes occidentalis*	Rosaceae	Winter forb	18
4	*Avena barbata*	Poaceae	Annual grass	12
5	*Briza minor*	Poaceae	Annual grass	25
6	*Brodiaea elegans*	Asparagaceae	Bulb	16
7	*Bromus carinatus*	Poaceae	Perennial grass	77
8	*Bromus diandrus*	Poaceae	Annual grass	26
9	*Bromus hordeaceus*	Poaceae	Annual grass	39
10	*Bromus madritensis*	Poaceae	Annual grass	5
11	*Bromus tectorum*	Poaceae	Annual grass	67
12	*Cardamine oligosperma*	Brassicaceae	Spring forb	23
13	*Castilleja attenuata*	Orobanchaceae	Spring forb	12
14	*Cerastium glomeratum*	Caryophyllaceae	Spring forb	28
15	*Cirsium occidentale*	Asteraceae	Perennial forb	10
16	*Clarkia purpurea*	Onagraceae	Spring forb	27
17	*Convolvulus arvensis*	Convolvulaceae	Perennial forb	24
18	*Cynosurus echinatus*	Poaceae	Annual grass	32
19	*Danthonia californica*	Poaceae	Perennial grass	38
20	*Daucus pusillus*	Apiaceae	Summer forb	14
21	*Dichelostemma capitatum*	Asparagaceae	Bulb	13
22	*Draba verna*	Brassicaceae	Spring forb	48
23	*Elymus glaucus*	Poaceae	Perennial grass	80
24	*Elymus multisetus*	Poaceae	Perennial grass	6
25	*Epilobium brachycarpum*	Onagraceae	Summer forb	75
26	*Eremocarpus setigerus*	Euphorbiaceae	Summer forb	12

Species ID	Name	Family	Functional group	#Occupied cells
27	*Eschscholzia californica*	Papaveraceae	Perennial forb	32
28	*Galium parisiense*	Rubiaceae	Spring forb	4
29	*Gastridium ventricosum*	Poaceae	Annual grass	2
30	*Geranium dissectum*	Geraniaceae	Spring forb	18
31	*Hemizonia congesta*	Asteraceae	Spring forb	5
32	*Hypochaeris glabra*	Asteraceae	Summer forb	13
33	*Leptosiphon bicolor*	Polemoniaceae	Winter forb	21
34	*Lotus micranthus*	Fabaceae	Nitrogen-fixing forb	9
35	*Lupinus bicolor*	Fabaceae	Nitrogen-fixing forb	31
36	*Madia gracilis*	Asteraceae	Summer forb	53
37	*Melanoplus devastator*	Acrididae	Generalist herbivore	7
38	*Micropus californicus*	Asteraceae	Winter forb	13
39	*Myosotis discolor*	Boraginaceae	Summer forb	29
40	*Navarretia divaricata*	Polemoniaceae	Spring forb	14
41	*Plagiobothrys nothofulvus*	Boraginaceae	Spring forb	11
42	*Platystemon californicus*	Papaveraceae	Spring forb	14
43	*Ranunculus occidentalis*	Ranunculaceae	Perennial forb	55
44	*Rumex acetosella*	Polygonaceae	Perennial forb	44
45	*Sanicula bipinnatifida*	Apiaceae	Perennial forb	21
46	*Sherardia arvensis*	Rubiaceae	Winter forb	23
47	*Stachys ajugoides*	Lamiaceae	Perennial forb	2
48	*Torilis arvensis*	Apiaceae	Summer forb	25
49	*Trichostema lanceolatum*	Lamiaceae	Summer forb	10
50	*Trifolium albopurpureum*	Fabaceae	Nitrogen-fixing forb	8
51	*Trifolium bifidum*	Fabaceae	Nitrogen-fixing forb	10
52	*Trifolium microcephalum*	Fabaceae	Nitrogen-fixing forb	49
53	*Trifolium willdenovii*	Fabaceae	Nitrogen-fixing forb	25
54	*Vulpia myuros*	Poaceae	Annual grass	36

Table S2: Climate variables at the Angelo Coast Range Reserve, California (39° 44′ 17.7″ N, 123° 37′ 48.4″ W).

Climate variable	2010
Maximum temperature of the warmest month	27.56°C
Minimum temperature of the coldest month	2.10°C
Annual precipitation	2036.41mm
Precipitation of the driest quarter	36.47mm
Mean temperature of the wettest quarter	8°C
Temperature seasonality (standard deviation × 100)	444
Precipitation seasonality (coefficient of variation)	84

Table S3: Model performance with original compression models and "reverse" versions

	Model	#Positive	#Negative	Δ_{M} Rank	$\Delta\%_{\mathrm{M}}$ Rank	Model	Δ_{M} Rank	$\Delta\%_{\mathrm{M}}$ Rank
maxSens threshold	ALL	40	12	4	3	reverse	1	4
	SHS BI	38	9	6	5	reverse	2	5
	SHS BI +	38	0	8	7	reverse	8	8
	SHS BI –	32	9	5	6	reverse	3	3
	SHS	32	0	7	8	reverse	6	7
	BI	6	9	2	2	reverse	4	2
	BI +	6	0	3	4	reverse	7	6
	BI –	0	9	1	1	reverse	5	1
maxSSS threshold	ALL	40	12	1	2	reverse	1	4
	SHS BI	38	9	2	4	reverse	2	5
	SHS BI +	38	0	4	6	reverse	4	6
	SHS BI –	32	9	3	3	reverse	3	2
	SHS	32	0	5	5	reverse	5	3
	BI	6	9	6	7	reverse	6	7
	BI +	6	0	8	8	reverse	8	8
	BI –	0	9	7	1	reverse	7	1

Table S4: Model performance when modelling different sets of interspecific relationships in species distribution models; absolute and percentage changes in compression and corresponding ranking (R) among compression models

		All interspecific relationships				Only shared habitat suitability				Only biotic interactions			
	Model	Δ_M	R	$\Delta\%_M$	R	Δ_M	R	$\Delta\%_M$	R	Δ_M	R	$\Delta\%_M$	R
maxSens threshold	ALL	-0.008	4	-2.8%	3	0.013	5	4.2%	5	-0.013	6	-4.2%	6
	SHS BI	-0.011	6	-3.8%	5	0.014	4	4.6%	4	-0.011	5	-3.8%	4
	SHS BI +	-0.016	8	-5.9%	7	0.015	2	5.5%	2	-0.018	7	-6.6%	7
	SHS BI −	-0.011	5	-3.8%	6	0.014	3	5.0%	3	-0.011	4	-3.8%	5
	SHS	-0.016	7	-6.2%	8	0.015	1	5.9%	1	-0.018	8	-7.0%	8
	BI	0.003	2	3.7%	2	-0.002	8	-2.2%	7	0.007	2	7.7%	2
	BI +	-0.001	3	-3.2%	4	0.000	6	0.0%	6	-0.001	3	-1.9%	3
	BI −	0.005	1	9.7%	1	-0.002	7	-3.6%	8	0.008	1	16.2%	1
maxSSS threshold	ALL	0.041	1	16.1%	2	0.034	2	13.2%	3	-0.001	4	-0.3%	4
	SHS BI	0.037	2	14.7%	4	0.033	2	13.1%	4	0.000	3	0.1%	3
	SHS BI +	0.029	4	12.5%	6	0.028	5	12.2%	5	-0.006	6	-2.4%	5
	SHS BI −	0.035	3	15.8%	3	0.038	1	17.2%	1	-0.006	7	-2.7%	6
	SHS	0.028	5	13.7%	5	0.033	3	16.2%	2	-0.010	8	-4.7%	7
	BI	0.008	6	9.6%	7	-0.002	7	-2.8%	7	0.006	1	7.2%	2
	BI +	-0.001	8	-1.2%	8	-0.004	8	-8.2%	8	0.006	2	11.0%	1
	BI −	0.007	7	20.9%	1	0.002	6	5.3%	6	-0.003	5	-7.9%	8

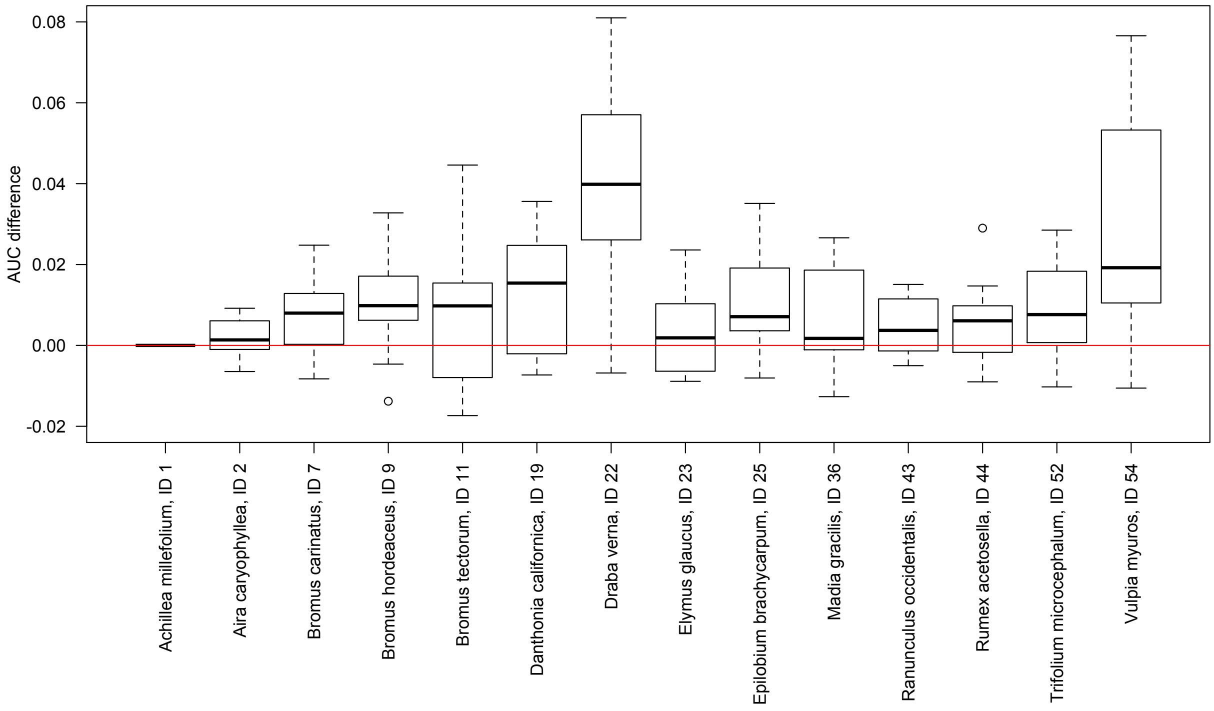

Figure S1: Increases and decreases in AUC scores for the 14 focal species when including all interspecific relationships in species distribution models (SDMs). For each focal species, we calculated the difference in the AUC score when using posteriors (output of an SDM with interspecific relationships) and when using priors (output of an SDM without interspecific relationships) for the same partition of data into training and test sets. Paired differences in AUC were calculated for 20 random partitions for each focal species. Box plots show interquartile range, median, minimum and maximum values of paired differences in AUC. If the difference in AUC is consistently greater than zero, then the Bayesian network is valuable and including the particular interspecific relationships in SDMs improves estimates of species geographical ranges.

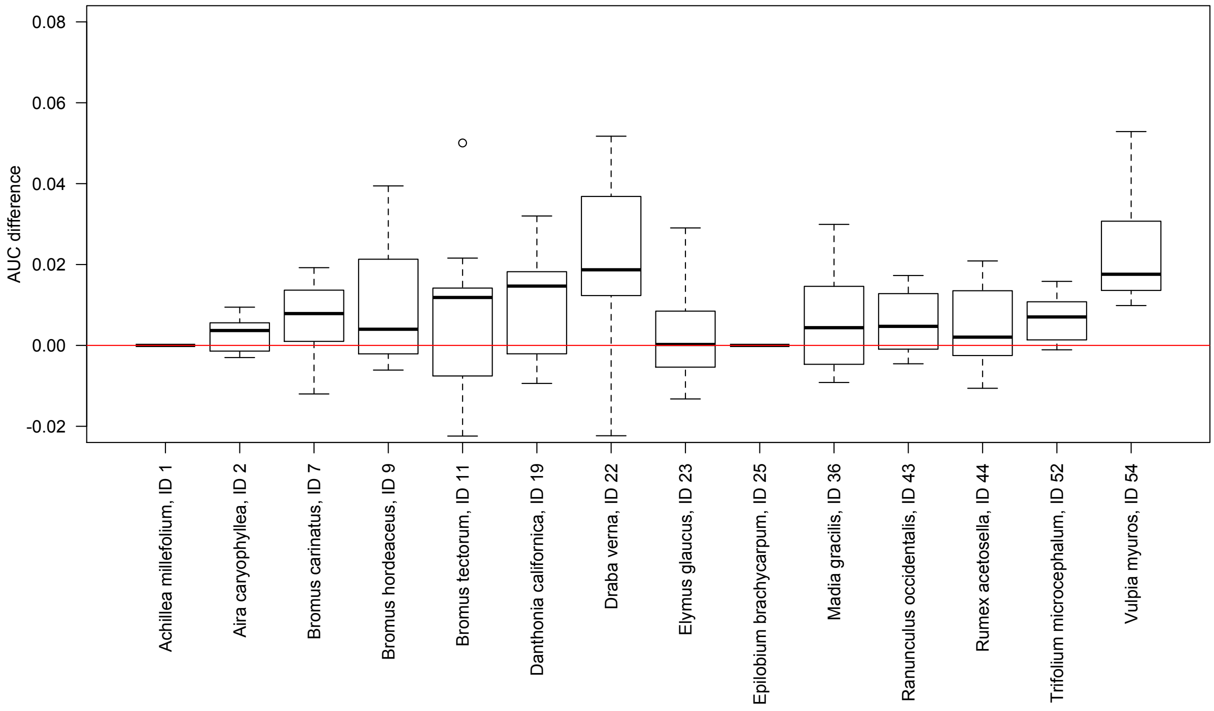

Figure S2: Increases and decreases in AUC scores for the 14 focal species when including only shared habitat suitability relationships in species distribution models (SDMs). For each focal species, we calculated the difference in the AUC score when using posteriors (output of an SDM with interspecific relationships) and when using priors (output of an SDM without interspecific relationships) for the same partition of data into training and test sets. Paired differences in AUC were calculated for 20 random partitions for each focal species. Box plots show interquartile range, median, minimum and maximum values of paired differences in AUC. If the difference in AUC is consistently greater than zero, then the Bayesian network is valuable and including the particular interspecific relationships in SDMs improves estimates of species geographical ranges.

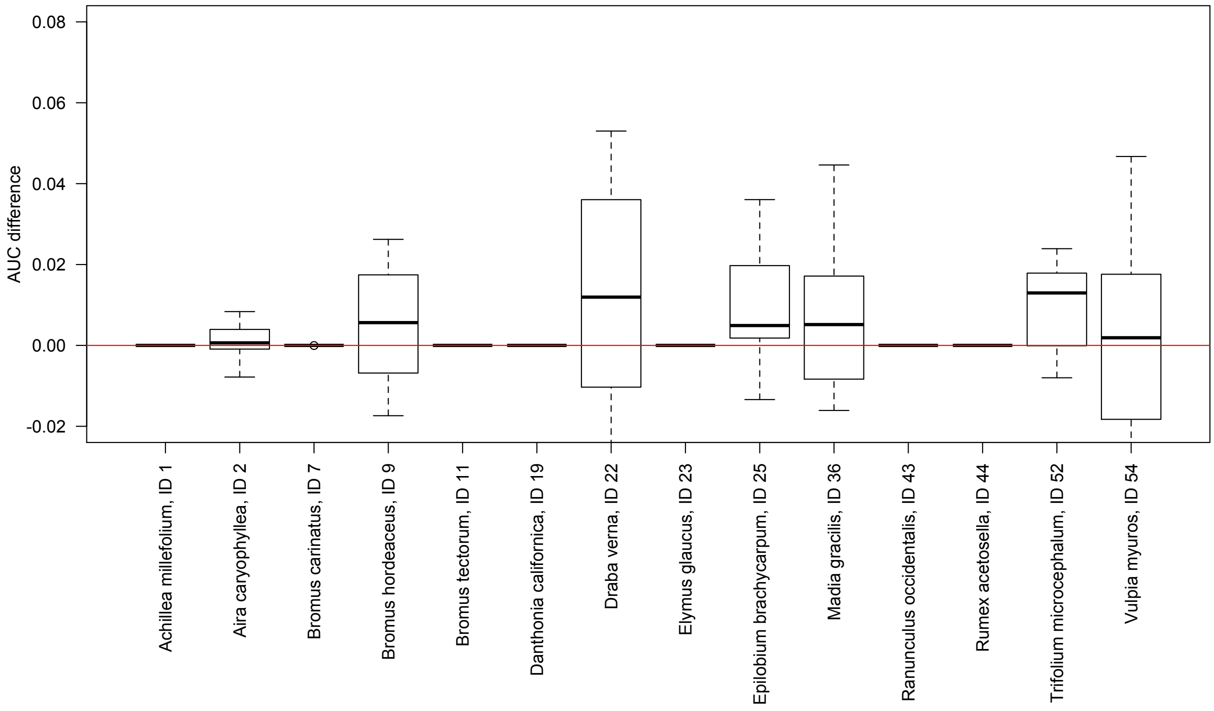

Figure S3: Increases and decreases in AUC scores for the 14 focal species when including only biotic interactions in species distribution models (SDMs). For each focal species, we calculated the difference in the AUC score when using posteriors (output of an SDM with interspecific relationships) and when using priors (output of an SDM without interspecific relationships) for the same partition of data into training and test sets. Paired differences in AUC were calculated for 20 random partitions for each focal species. Box plots show interquartile range, median, minimum and maximum values of paired differences in AUC. If the difference in AUC is consistently greater than zero, then the Bayesian network is valuable and including the particular interspecific relationships in SDMs improves estimates of species geographical ranges.
